# Evaluation of graphene oxide, chitosan and their complex as antibacterial agents and anticancer apoptotic effect on HeLa cell line

**DOI:** 10.3389/fmicb.2022.922324

**Published:** 2022-10-04

**Authors:** Noha M. Ashry, Halla E. K. El Bahgy, Abdelkader Mohamed, Nouf H. Alsubhi, Ghadeer I. Alrefaei, Najat Binothman, Mona Alharbi, Samy Selim, Mohammed S. Almuhayawi, Mohanned T. Alharbi, Mohammed K. Nagshabandi, Ahmed M. Saad, Mohamed T. El-Saadony, Basel Sitohy

**Affiliations:** ^1^Department of Agriculture Microbiology, Faculty of Agriculture, Benha University, Qalubia, Egypt; ^2^Department of Veterinary Hygiene and Management, Faculty of Veterinary Medicine, Benha University, Banha, Egypt; ^3^Department of Soil and Water Research, Nuclear Research Center, Egyptian Atomic Energy Authority, Abou Zaabl, Egypt; ^4^Department of Biological Sciences, College of Science and Arts, King Abdulaziz University, Rabigh, Saudi Arabia; ^5^Department of Biology, College of Science, University of Jeddah, Jeddah, Saudi Arabia; ^6^Department of Chemistry, College of Sciences and Arts, King Abdulaziz University, Rabigh, Saudi Arabia; ^7^Department of Biochemistry, College of Science, King Saud University, Riyadh, Saudi Arabia; ^8^Department of Clinical Laboratory Sciences, College of Applied Medical Sciences, Jouf University, Sakaka, Saudi Arabia; ^9^Department of Medical Microbiology and Parasitology, Faculty of Medicine, King Abdulaziz University, Jeddah, Saudi Arabia; ^10^Department of Medical Microbiology and Parasitology, Faculty of Medicine, University of Jeddah, Jeddah, Saudi Arabia; ^11^Department of Biochemistry, Faculty of Agriculture Zagazig University, Zagazig, Egypt; ^12^Department of Agricultural Microbiology, Faculty of Agriculture, Zagazig University, Zagazig, Egypt; ^13^Department of Radiation Sciences, Oncology, Umeå University, Umeå, Sweden; ^14^Department of Clinical Microbiology, Infection and Immunology, Umeå University, Umeå, Sweden

**Keywords:** chitosan, graphene oxide, complex, antibacterial, anticancer, HeLa cells, P53 gene

## Abstract

Cancer and bacterial infection are the most serious problems threatening people's lives worldwide. However, the overuse of antibiotics as antibacterial and anticancer treatments can cause side effects and lead to drug-resistant bacteria. Therefore, developing natural materials with excellent antibacterial and anticancer activity is of great importance. In this study, different concentrations of chitosan (CS), graphene oxide (GO), and graphene oxide-chitosan composite (GO-CS) were tested to inhibit the bacterial growth of gram-positive (*Bacillus cereus* MG257494.1) and gram-negative (*Pseudomonas aeruginosa* PAO1). Moreover, we used the most efficient natural antibacterial material as an anticancer treatment. The zeta potential is a vital factor for antibacterial and anticancer mechanism, at pH 3–7, the zeta potential of chitosan was positive while at pH 7–12 were negative, however, the zeta potential for GO was negative at all pH values, which (*p* < 0.05) increased in the GO-CS composite. Chitosan concentrations (0.2 and 1.5%) exhibited antibacterial activity against BC with inhibition zone diameters of 4 and 12 mm, respectively, and against PAO1 with 2 and 10 mm, respectively. Treating BC and PAO1 with GO:CS (1:2) and GO:CS (1:1) gave a larger (*p* < 0.05) inhibition zone diameter. The viability and proliferation of HeLa cells treated with chitosan were significantly decreased (*p* < 0.05) from 95.3% at 0% to 12.93%, 10.33%, and 5.93% at 0.2%, 0.4%, and 0.60% concentrations of chitosan, respectively. Furthermore, CS treatment increased the activity of the P53 protein, which serves as a tumor suppressor. This study suggests that chitosan is effective as an antibacterial and may be useful for cancer treatment.

## Introduction

Pathogens remain a serious health hazard, resulting in many annual deaths (Vijayalakshmi et al., 2016). The infection risk caused by microorganisms has recently become a major concern in the pharmacological, clinical, and diet industries. Cancer is also the second cause of death globally. It starts with abnormal uncontrollable cell growth in any body cell and then spreads to other body parts. The latter process is called metastasizing and is the major cause of death from cancer (WHO, 2020). Cervical cancer is epithelial cancer ranked as the world's most common cancer in women (Shehata, 2005). The scientific community has developed new and effective antibacterial materials through multiple strategies to improve protection against pathogenic bacteria. Among various materials, graphene has excellent conductivity and good thermal, optical, and mechanical properties (Barbolina et al., 2016). According to the chemical structure, Sun et al. (2020) define graphene oxide (GO) as a honeycomb-structured carbon compound with hydroxide, carbonyl, and carboxylic moieties at its basal plane. These functionalities are decreased in reduced graphene oxide, and this boosts the intrinsic properties of GO (Jilani et al., 2019).

Previous studies have shown that GO is an antimicrobial agent (Chen et al., 2013; Díez-Pascual, 2020; Menazea and Ahmed, 2020), and its potential antibacterial properties have been linked to cell wrapping, sharp-edged contact, oxidative stress, and phospholipid damage extraction. Other studies showed that it was not toxic to the bacteria (Das et al., 2011; Ruiz et al., 2011). Although graphene-based materials and graphene are used in many fields, there is a point of conflict about their antibacterial activities (Mohammed et al., 2020).

Chitosan (CS) is a polycationic compound consisting of N-acetyl glucosamine and glucosamine that has an antimicrobial effect on gram-positive (G+) and gram-negative (G–) bacteria by disrupting the microbial cell, then its death (Vijayalakshmi et al., 2016; Bhattacharjee et al., 2020). The difference in charges between positively charged CS molecules and negatively charged microbial cell membranes (CM) makes CS an effective antimicrobial substance (Sundar et al., 2014). It is known that CS has different pharmacological effects, such as antibacterial (Rashki et al., 2021) and antitumor (Gibot et al., 2015). Although the mechanism of how chitosan interacts with cancer cells remains unclear, previous studies have suggested several possible mechanisms, extracellularly binding of bigger-molecule CS to the CM, endocytosis, or internalization of CS nanoparticles (NPs) (Huang et al., 2004). Yang et al. (2009) suggested that these mechanisms might be set off by an ionic reaction between positively charged CS molecules and negatively charged cancer CM, which triggers signaling pathways that lead to apoptosis or autophagy.

Moreover, CS has anticancer activity against cervical and epithelial cancers. Its anticancer activity may be through apoptosis, and the cell cycle stops at the G0/G1 phase by lowering the cell viability and activating caspase-3 (Prasad et al., 2019; Chang et al., 2021). Deepika et al. (2019) found that CS compounds increase the expression of the P53 gene while decreasing the expression of the Bcl-2 protein.

Recently, some trials have been conducted to produce complexes of graphene oxide with nanomaterials to valorize their stability and activities. A mixture of GO with CS has been used as an antibacterial (Kyzas et al., 2014; Ordikhani et al., 2015), wherein the CS functional groups may interact with the epoxide, carboxyl, and hydroxyl groups patterned at the base level and GO edges. Although several studies used CS and GO as antibacterial materials, Jilani et al. (2019) developed a combination between GO and Cu–ZnO nanoparticles and found that the surface electric charge conducted by GO or rGO enhanced the dielectric constant of Cu–ZnO nanoparticles.

Furthermore, Oves et al. (2020a) discovered that graphitic C3N4@ Polyaniline Composites are an effective antimicrobial agent and have high stability at temperatures above 100°C. Furthermore, Oves et al. (2020b) found that graphene-based nano-zinc oxide nanoparticles (ZnO-NPs) showed a wide range of antimicrobial activity against methicillin-resistant *Staphylococcus aureus* (MRSA). This activity was enhanced >5 times when combined with curcumin. There is a lack of studies using different ratios of GO and CS mixtures. They were also due to the disagreement over whether GO could be used as an antibacterial agent or not. Therefore, the main objective was to determine the antimicrobial effects of CS, GO, and their combination against G+ and G– bacteria. Then, we examined the concentrations of the most efficient substance that showed high results as an antibacterial agent for its ability to have anticancer activity on the HeLa cell line to reach the lowest anticancer concentration with fewer side effects.

## Materials and methods

### Bacterial strains and culture features

*Pseudomonas aeruginosa* PAO1 was acquired from the College of Resources and Environment, Huazhong Agricultural University, China. *Bacillus cereus* MG257494.1 (BC) was acquired from the Microbiology Department, Faculty of Agriculture, Benha University, Qalyubia Governorate, Egypt. Cultures in Luria-Bertani (LB) broth medium containing 50% glycerol as a frozen stock (−80°C, RS Biotech freezer, Richmond Scientific Ltd., Lancashire, PR6 0RE, Great Britain). The strains were developed in LB broth medium in a rotary incubator at 37°C overnight and then centrifuged at 6,000 rpm for 5 min to harvest the bacterial cells. The pellets were washed 3 times with deionized water and rehung in deionized water. The suspension (susp.) was diluted to the desired concentration of 1 × 10^6^ colony-forming units (CFUml^−1^).

### Physiochemical characterization

GO was purchased from Time Nano Chengdu Organic Chemicals (catalog number TNGO). The purity of GO was >99.5 wt., with a thickness of 0.55–1.2 nm and a diameter of 0.5–3 μm. CS was obtained from Aladdin Reagent Database Co. (Shanghai, China). The zeta potentials of GO, CS, GO:CS composite, and two bacterial strains were analyzed at different pH (2, 4, 6, 8, and 10) using the Zeta plus 90 potentiometers (Brookhaven, USA).

### Preparation of CS and GO concentrations

Chitosan stock SOLN (2%) (w/v) was prepared using 1% aqueous acetic acid and then diluted to a final concentration of 0.2–1.5%. Different GO concentrations that ranged from 100 to 700 μg ml^−^ were prepared from 1,000 μg ml^−1^ GO stock solution (SOLN). Deionized distilled water was utilized as a control treatment. Furthermore, for BC and PAO1, different mixture ratios of GO to CS (2:1, 1:1, and 1:2) were prepared using GO (700 μg ml^−1^) and CS (0.6 and 0.8%), respectively.

### Biological activities of CS, GO, and their composites

#### Antibacterial

The antibacterial effect of CS, GO, and their mixture was examined against gram-negative PAO1 and gram-positive BC using turbidity measurement and the agar diffusion methods (Hong et al., 2007; Jiang et al., 2013) and cell viability loss determination (Chen et al., 2013).

To determine the turbidity of the bacteria, 200 μl of the diluted cell suspension of each bacterium (OD600 = 0.5) was mixed with 20 μl of various starting GO concentrations (100, 200, 300, 400, 500, 600, and 700 mg ml^−1^), various starting levels of CS (0.2, 0.4, 0.6, 0.8, 1, and 1.5%), and the previous different ratios of GO:CS. The control treatment was prepared by adding 200 μl of the cell suspension to 20 μl of deionized sterilized water. The mixtures were then incubated at 30°C for 2 h with gentle mixing. Then, 2 ml of LB medium was added to the mixture in 5 ml tubes, and the tubes were kept on a rotary shaker at 120 rpm and 30°C. The optical density value (OD) at a wavelength of 600 nm was estimated at initial, 12, 14, 16, and 18 h. OD values were plotted against time to create bacterial curves. The three-triplicate setup was prepared for all treatments.

For the agar diffusion method, 100 μl of bacterial suspension was added to the surface of the LB solid culture medium and spread well. Sterile filter paper disks (6 mm in diameter) immersed with 20 μl of various levels of the previously tested substrates were placed on the surface of each LB plate using sterile forceps. Saturated water disks were used as a control. The plates were kept under aerobic conditions at 37°C for 24 h. The inhibition zone diameter was assessed after 24 h based on the average diameter of the clear area using a ruler or caliper. Three replicate plates were used for each concentration.

As for the cell viability loss determination, 200 μl of the bacterial suspension (OD600 = 0.5) was kept with 20 μl of the same previously used concentrations for 2 h with shaking and then diluted to a dilution factor of 10^−6^; then 20 μl of bacterial dilution was streaked on LB plates and kept for 24 h at 30°C. Colonies were counted from each treatment, and antibacterial activity was expressed as a function of loss of cell viability. All treatments were triplicates.


(1) $$\begin{array}{l} \%\text{ cell death}\\ =\frac{\text{cell No of control }-\text{ cell No of treated samples}}{\text{cell No of control}}x100 \end{array}$$

#### Anticancer

##### Cells

The human cervical tumor cell line, HeLa cell, was acquired from the Cell Bank of Shanghai. The cells were grown in Dulbecco's modified Eagle medium (DMEM), including 10% fetal bovine serum and 100 units/ml penicillin/streptomycin. The cells were kept at 37°C and 5% CO_2_.

##### Cell viability assay

The cytotoxic effect of chitosan on HeLa cells was determined using cell counting kit-8 (CCK-8) (Agilent Technologies, Santa Clara, CA 95051, United States). HeLa cells were seated in 96-well plates at 10^4^ cells/well density and incubated overnight to permit cell attachment. Then the media were discarded, and the cell layer was washed with phosphate-buffered saline (PBS). Various levels of chitosan (0%, 0.2%, 0.4%, and 0.6%) dissolved in DMEM media were added to the washed cell layer and then incubated for 24 h. Each well received 10 μl of CCK SOLN, and the plate was left in the dark for 4 h. The plate was measured at 450 nm by a microplate reader (Agilent Technologies, Santa Clara, CA 95051, United States).


(2) $$\begin{array}{l} Cell\;viability\;=\;\frac{\left(OD\;treated-OD\;blank\right)}{\left(OD\;Neg\;control-OD\;blank\right)} \end{array}$$

##### Cell apoptosis assay

HeLa cells (1 × 10^6^ cells/well) were cultured in 6-well plates and kept overnight to permit cell attachment. After adding chitosan at 0%, 0.20%, and 0.40% for 24 h, cells were rinsed 3 times with PBS. Samples were obtained according to the manufacturer's guidelines using the Annexin V-FITC Apoptosis Detection Kit (Huazhong Agricultural University, China). The obtained cells were analyzed on the Epics Altra II flow cytometer. The apoptosis rate was estimated by averaging the quantities of early and late apoptotic cells. The test was presented in 3 replicates.

##### Reverse transcription and quantitative polymerase chain reaction

Total RNA was obtained from chitosan treated and untreated HeLa cell lines using EZNA reagent (OMEGA bio-TEKRE agent) and cDNA was reverse transcribed from RNA using Superscript II reverse transcriptase, according to the manufacturer's recommendations. The below primer pairs for target genes and B-actin were selected from the primer bank website.

P53 oligonucleotide primers were F 5′CCTCAGCATCTTATCCGAGTGG3′ and R5′TGGATGGTGGTACAGTCAGAGC3′ (ACC. NO: NM_000546), caspase-3 primers were F 5′GGAAGCGAATCAATGGACTCTGG3′ and R 5′GCATCGACATCTGTACCAGACC3′ (ACC. NO: NM_004346), caspase-9 primers were F 5′GTTTGAGGACCTTCGACCAGCT3′ and R 5′CAACGTACCAGGAGCCACTCTT3′ (ACC. NO: NM_001229), BCL_2 primers were F 5′ATCGCCCTGTGGATGACTGAGT 3 and R 5′GCCAGGAGAAATCAAACAGAGGC 3′ (ACC. NO: BC027258, NM_000633, and NM_000657), and B-actin primers were F 5′CACCATTGGCAATGAGCGGTTC3 and R 5′AGGTCTTTGCGGATGTCCACGT3′ (ACC. NO: NM_001101). The primer was obtained from AUGCT DNA-SYN Biotechnology Synthesis Lab, China.

SYBER green master mix (chamQtm SYBR^®^ qPCR Master mix, Vazyme Biotech Co., China) was used for qPCR assays. The qPCR program was set as follows: 95°C for 30 s, followed by 40 cycles at 95°C for 10 s, 60°C for 30 s, and three melting steps of 95°C for 15 s, 60°C for 60 s, and 95°C for 15 s, with a final dissociation curve. Each reaction was carried out three times.

### Statistical evaluation

Findings and outcomes were tested using SPSS version 20, using a one-way analysis of variance (ANOVA), and the differences appeared statistically significant at a *p* ≤ 0.05.

## Results

### Physiochemical properties

#### Zeta potential analysis

**Figure 1:**
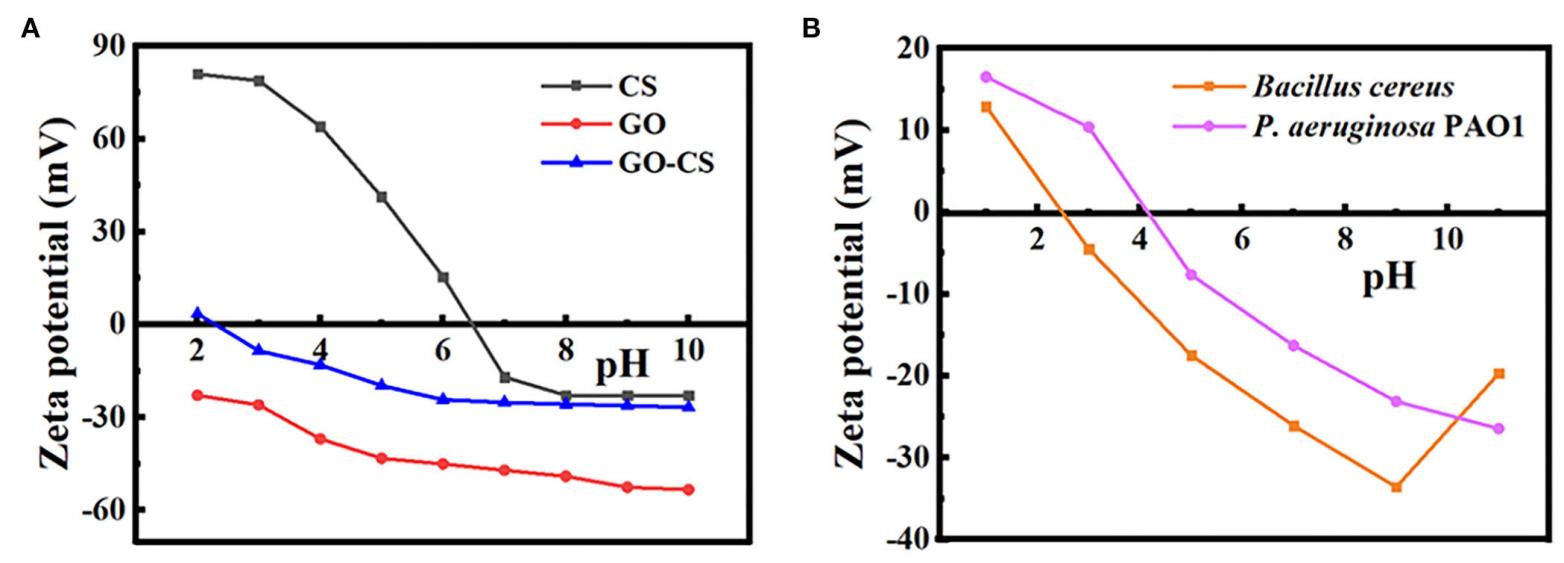
**(A)** Zeta potential of chitosan, grapheneoxide, CS-GO. **(B)** Zeta potential of *Bacillus cereus, Pseudomonas aeruginosa*.

The zeta potentials of GO, CS, GO-CS composite, and two bacterial strains were analyzed at different pH values (2, 4, 6, 8, and 10), characterized, and shown in Figure 1. At pH 5–9, the zeta potentials for PAO1 and BC were negative. The zeta potential of chitosan was positive in acidic conditions, while it was negative in alkaline conditions. At pH 3–7, the zeta potential of chitosan SOLN reduced linearly. While increasing the pH value from 7 to 12, the negative zeta potential does not decrease significantly (*p* > 0.05). The zeta potential for GO was negative at all pH values. With increasing pH values, there was no significant increase (*p* > 0.05) in the negative zeta potential for GO-CS. While, after mixing GO with CS, the zeta potential significantly rises (*p* < 0.05) compared to GO individually.

### Antibacterial activity of CS, GO, and GO:CS composite

#### Turbidity

**Figure 2:**
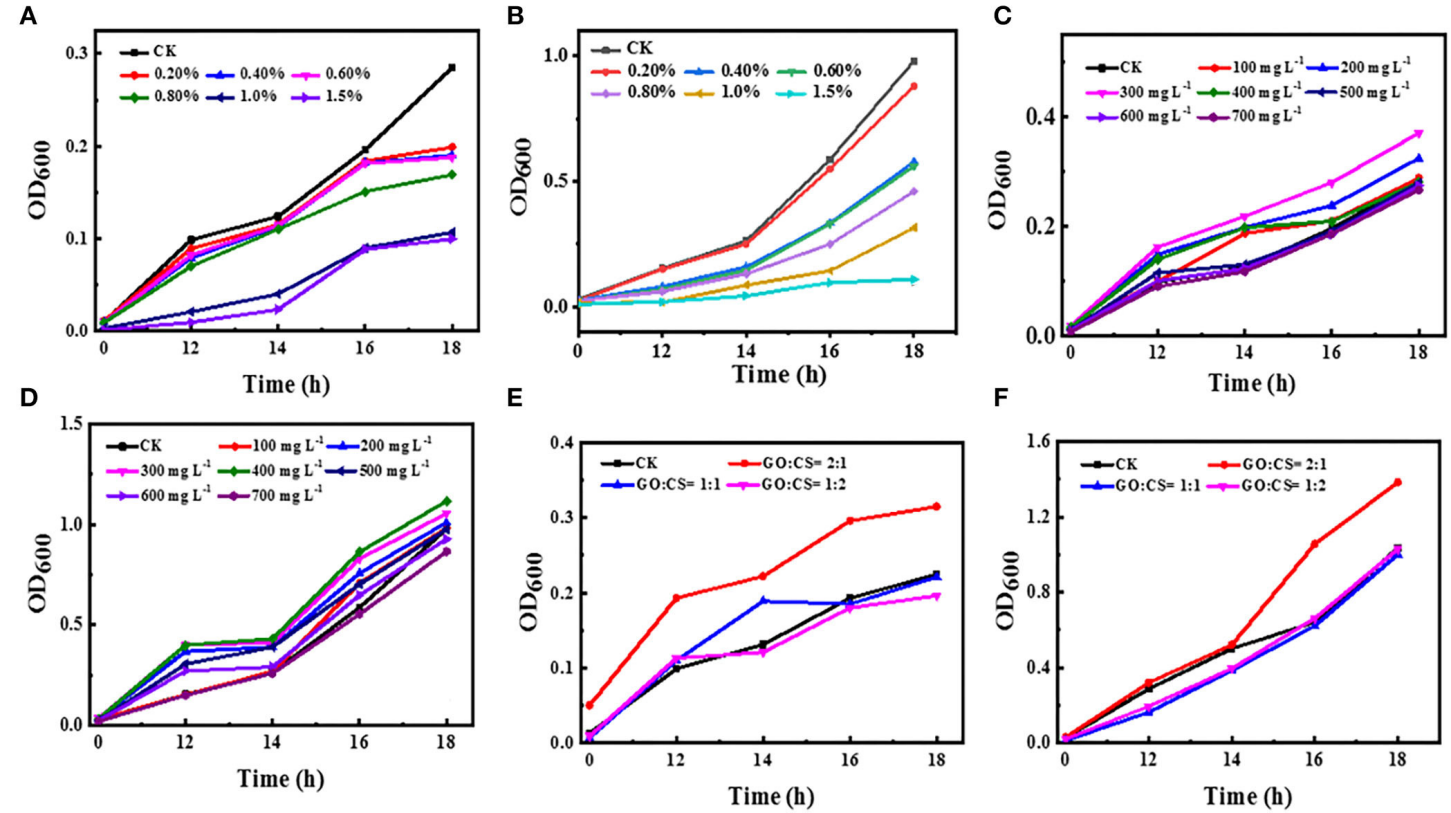
Effect of different concentrations of chitosan **(A,B)**, different concentrations of GO **(C,D)**, and different ratios of GO:CS **(E,F)**, respectively, compared to control (CK) on BC and PAO1.

The growth inhibitory effect of CS against BC and PAO1 was investigated by plotting the growth curve depending on turbidity (OD_600_ measurement) at different time points compared to vehicle control (Figures 2A,B). The turbidity of both bacterial strains significantly (*p* < 0.05) decreased with increasing chitosan concentrations, whereas, in the absence of CS, the turbidity increased in the function of time (control). Besides, CS significantly (*p* < 0.05) inhibits bacterial growth in the range of 0.2–1.5% compared to control. Additionally, with CS treatment, BC showed low turbidity than PAO1.

The antibacterial activity of GO with different concentrations against BC and PAO1 was also evaluated (Figures 2C,D). The OD value of the control group without graphene (CK) was initially 0.01 and 0.02 for BC and PAO1, respectively. After 18 h of incubation, its value reached 0.285 and 0.980 for BC and PAO1, respectively. Unpredictably, with the addition of GO, the OD_600_ value peaked at a concentration of 300 μg ml^−1^ (0.37) for BC and a concentration of 400 μg ml^−1^ (1.11) for PAO1. Then the OD value decreased thereafter with increasing the concentration. There is a significant difference (p>0.05) in growth inhibition compared to untreated bacteria (vehicle control), or there may be very slight inhibition when CO is used at a high concentration.

Regarding the GO-CS composite, all treatments showed increases in turbidity with increasing time. Surprisingly, when both bacterial strains were treated with GO:CS (2:1), the density of the bacteria was increased compared to the vehicle control. However, GO-CS (1:2) treatment exhibited a significant reduction (*p* < 0.05) in turbidity compared to the vehicle control and the other treatments. In contrast, PAO1 treatment with both GO:CS (1:1) or GO:CS (1:2) resulted in a similar decrease (*p* < 0.05) in turbidity (Figures 2E,F).

#### Inhibition zone measurement

**Figure 3:**
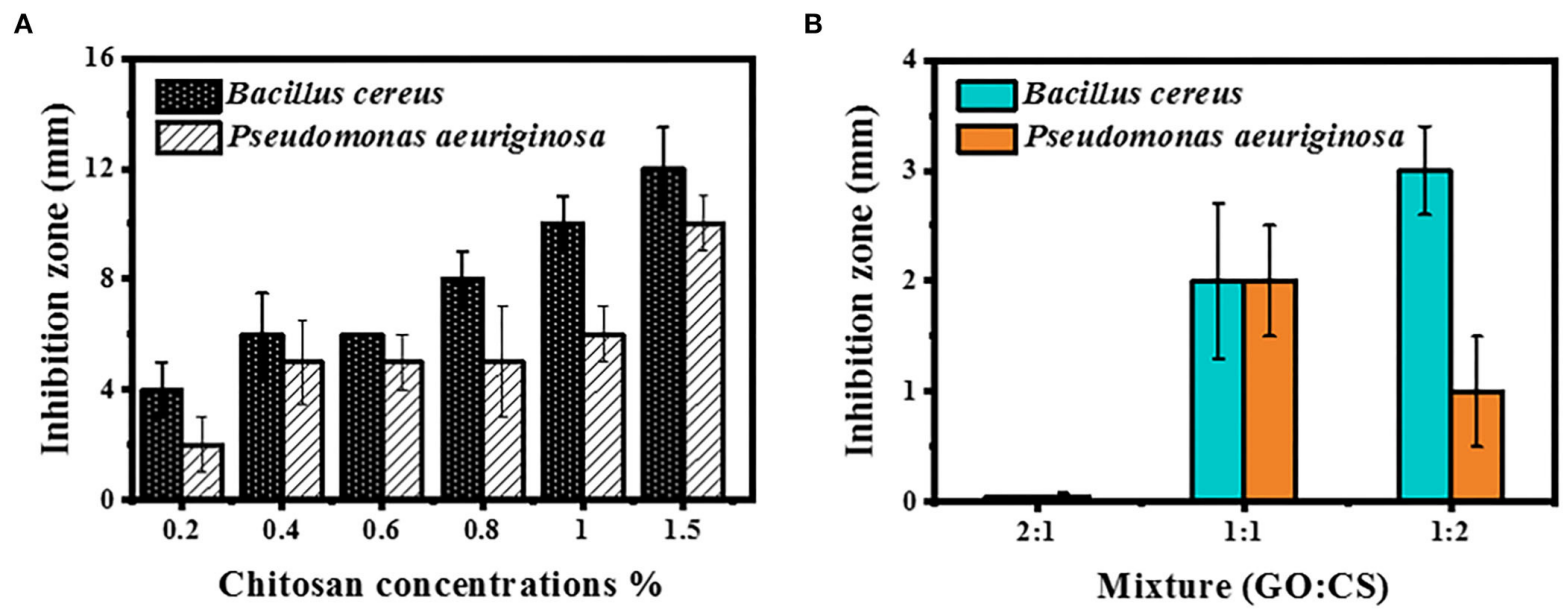
Inhibition zone measurement by BC and PAO1 at different concentrations of chitosan **(A)** and different ratios of GO:CS composite **(B)**.

**Figure 4:**
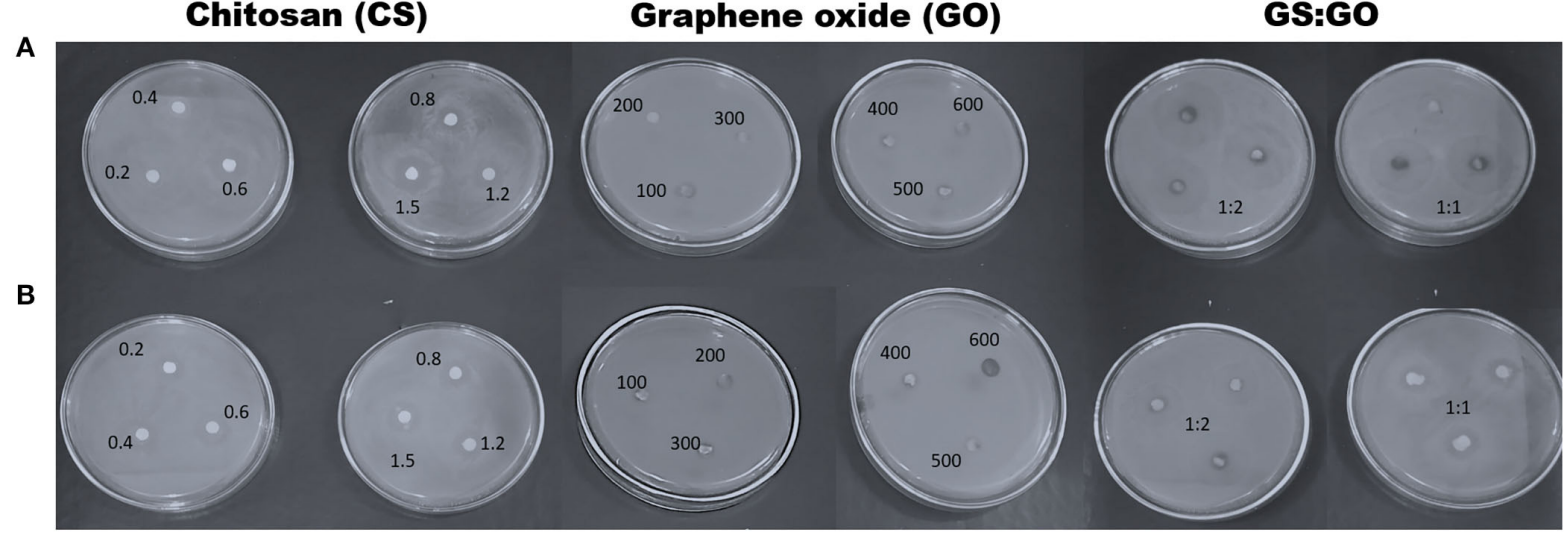
Inhibition zone diameters of chitosan, graphene oxide, and different combinations (1:1 and 1:2) against *Bacillus cereus*, BC **(A)**, and *Pseudomonas aeruginosa*, PAO1 **(B)**.

Antimicrobial activity at different concentrations of CS, GO, and their combinations was determined by disc diffusion (Figures 3A,B). The inhibition zone diameter increased by 1.5% when the chitosan concentrations were increased to their maximum concentrations. Chitosan activity against BC showed inhibition zone diameters of 4 and 12 mm at a concentration of 0.2 (the least concentration) and 1.5% (the highest concentration), respectively, while the inhibition zone diameter was 2 and 10 mm, respectively, for PAO1. Conversely, no inhibition zone was observed when both bacterial strains were handled with various levels of GO and GO:CS (2:1). Moreover, BC treatment with GO:CS (1:2) resulted in a larger (*p* < 0.05) inhibition zone diameter compared to treatment with GO:CS (1:1). PAO1 treatment with GO:CS (1:1) resulted in a higher (*p* < 0.05) inhibition zone diameter than that of GO:CS (1:2). The inhibition zones diameters are also shown in Figure 4.

#### Cell viability loss determination

**Figure 5:**
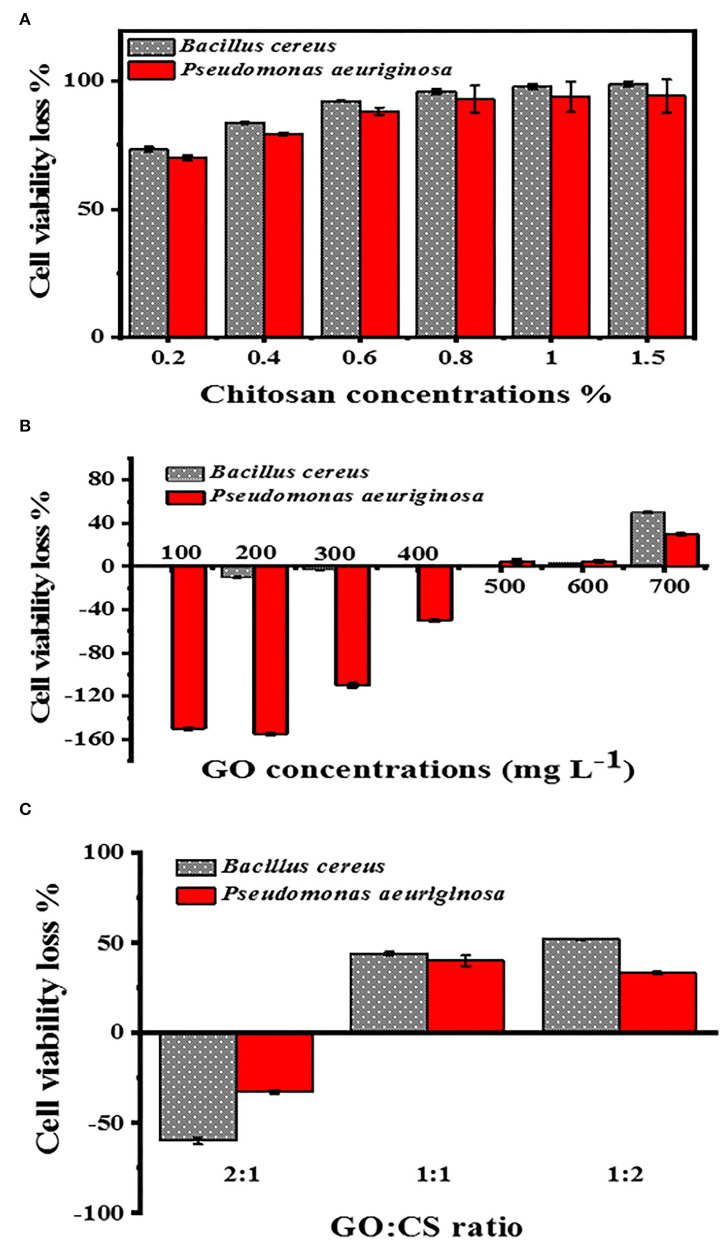
Cell viability loss determination at different concentrations of CS **(A)**, with different concentrations of GO **(B)**, and different ratios of GO:CS **(C)**.

When the BC and PAO1 were exposed to CS concentration at 0.2 %, the lowest viability was 73.3 and 70%, respectively, but when the CS concentration was 1.5%, the minimum viability for BC and PAO1 was 99 and 94.33%, respectively (Figure 5A). Concerning the different concentrations of GO (Figure 5B), BC showed more (*p* < 0.05) rapid growth than the vehicle control (bacteria without GO) until the concentration of 300 μg ml^−1^ and then the number of bacteria became similar to the control. In comparison, it was found that at the concentrations of 600 and 700 μg ml^−1^, the cell viability loss for BC was 3.3 and 50%, respectively.

Nevertheless, in the case of PAO1, there was an elevation in the count of bacterial cells until a concentration of 400 μg ml^−1^. A very slight loss of viability cells was also obtained at GO levels of 500, 600, and 700 μg ml^−1^; they were 5, 5, and 30%, respectively. With the GO:CS combination at a ratio of 2:1, there was higher growth than the other ratios of both BC and PAO1. In contrast, their combination at a ratio of 1:2 showed a significant increase (*p* < 0.05) in cell viability loss of 52 and 33.3% for BC and PAO1, respectively (Figure 5C).

### Anticancer activity

**Figure 6:**
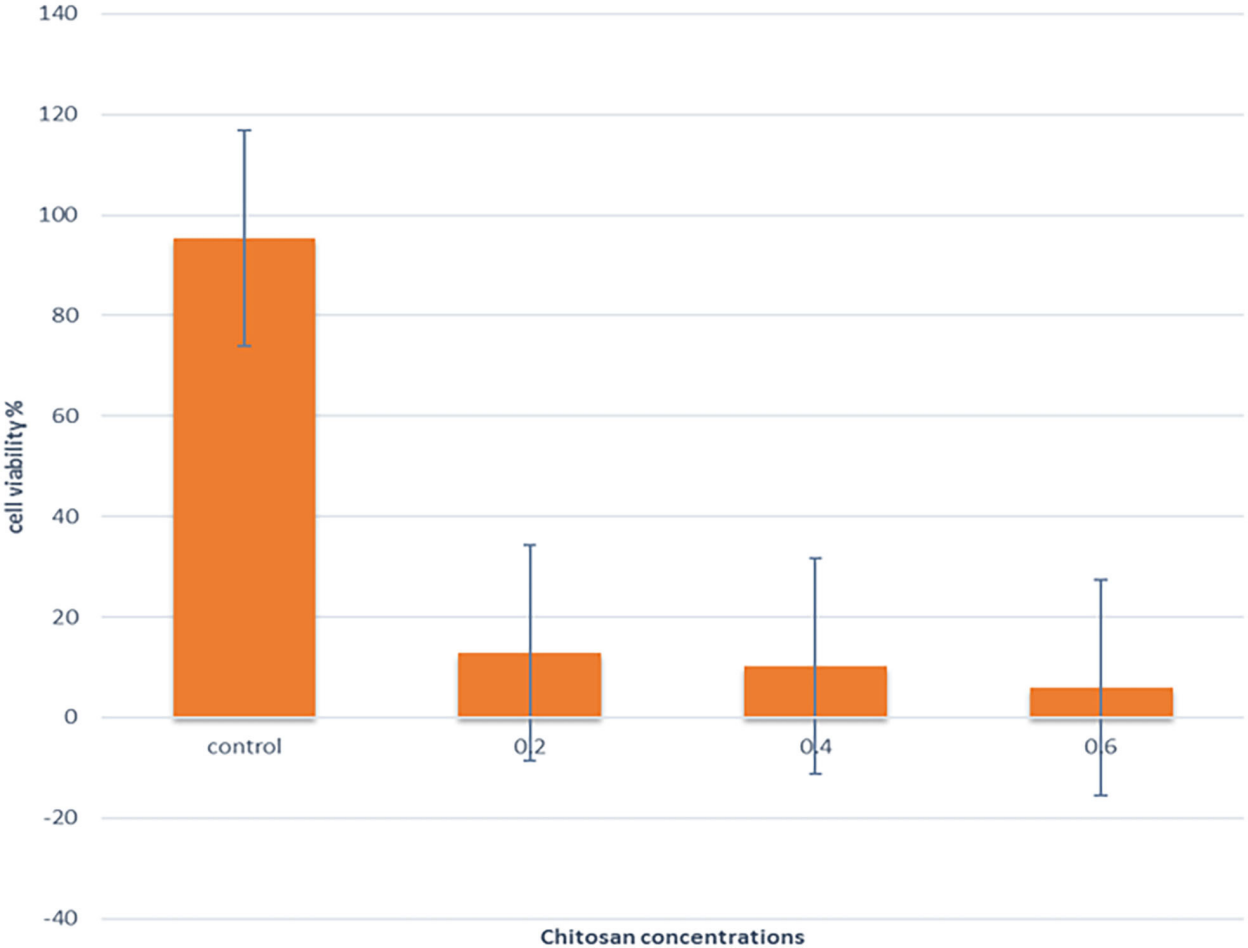
The effect of different concentrations of CS on the viability of the HeLa cell line was assessed by CCK-8.

Low concentrations of CS (0.2, 0.4, and 0.6%), which showed antibacterial efficacy, were selected to evaluate the anticancer effects. Figure 6 showed that the cell viability and cell proliferation of HeLa cells treated with chitosan were significantly decreased (*p* < 0.05) from 95.3% at 0% to 12.93%, 10.33, and 5.93% at 0.2%, 0.4%, and 0.60% concentrations of chitosan, respectively.

**Figure 7:**
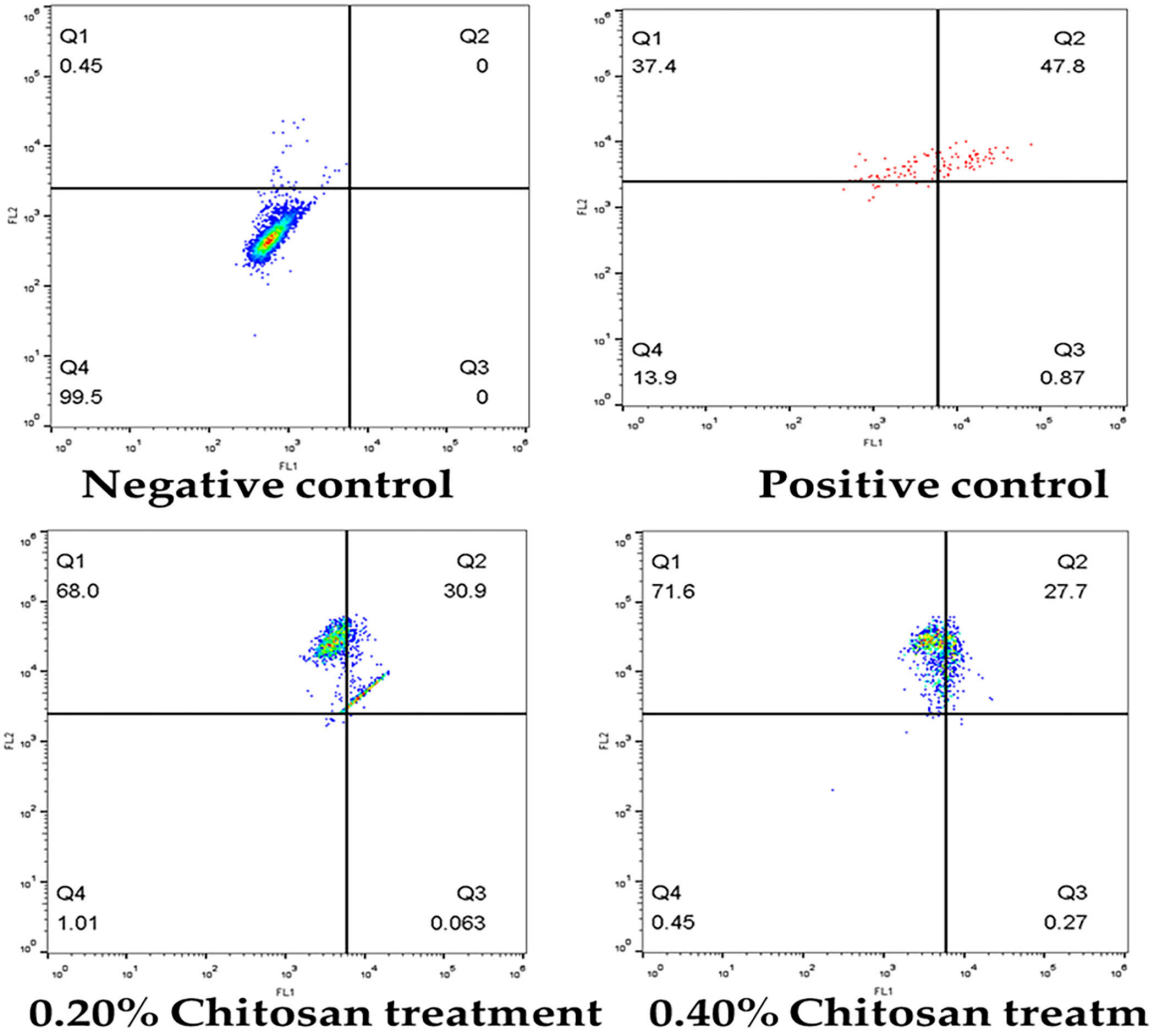
Flow cytometric analysis of apoptosis induction in HeLa cells treated with different concentrations of chitosan.

Figure 7 illustrates that the treatment of HeLa cells with different chitosan concentrations for 24 h resulted in a significant elevation in the apoptotic cell percentage and dead cells and a significant reduction in the viable cell percentage. Treating the HeLa cells with 0.2% for 24 h revealed a lowering of viability from 99.95 to 1.01%, and the apoptosis rate increased from 0 to 30.94%. The proportion of dead cells increased to 68.0%, compared with vehicle control. In addition, treating the HeLa cells with 0.4% for 24 h significantly decreased (*p* < 0.05) viability to 0.45%, increased (*p* < 0.05) early apoptosis to 0.27%, increased (*p* < 0.05) late apoptosis to 27.7%, and increased (*p* < 0.05) dead cells to 71.6%, relative to untreated controls.

The P53 gene was found to be significantly upregulated in treated Hela cells. In contrast, a significant decrease in the mRNA level of caspase-9 in Hela cells after treatment with chitosan, but the expression levels of *caspase3* and *Bcl2* in Hela cells were not changed (Table 1).

**Table 1:** Chitosan effects on the P53, caspase-3, caspase-9, and BCL_2 mRNA levels in HeLa cells after treatment with chitosan for 24 h.

	**Control group**	**Chitosan treatments**
P53	1.00 ± 0.00*	1.77 ± 0.15*
CASP9	1.00 ± 0.00*	0.37 ± 0.07*
CASP3	1.00 ± 0.00	1.13 ± 0.24
BCL2	1.00 ± 0.00	1.33 ± 0.56

*The relative quantification of the target gene by the delta-delta-Ct method was done usingthe Qiagen software.

**Figure 8:**
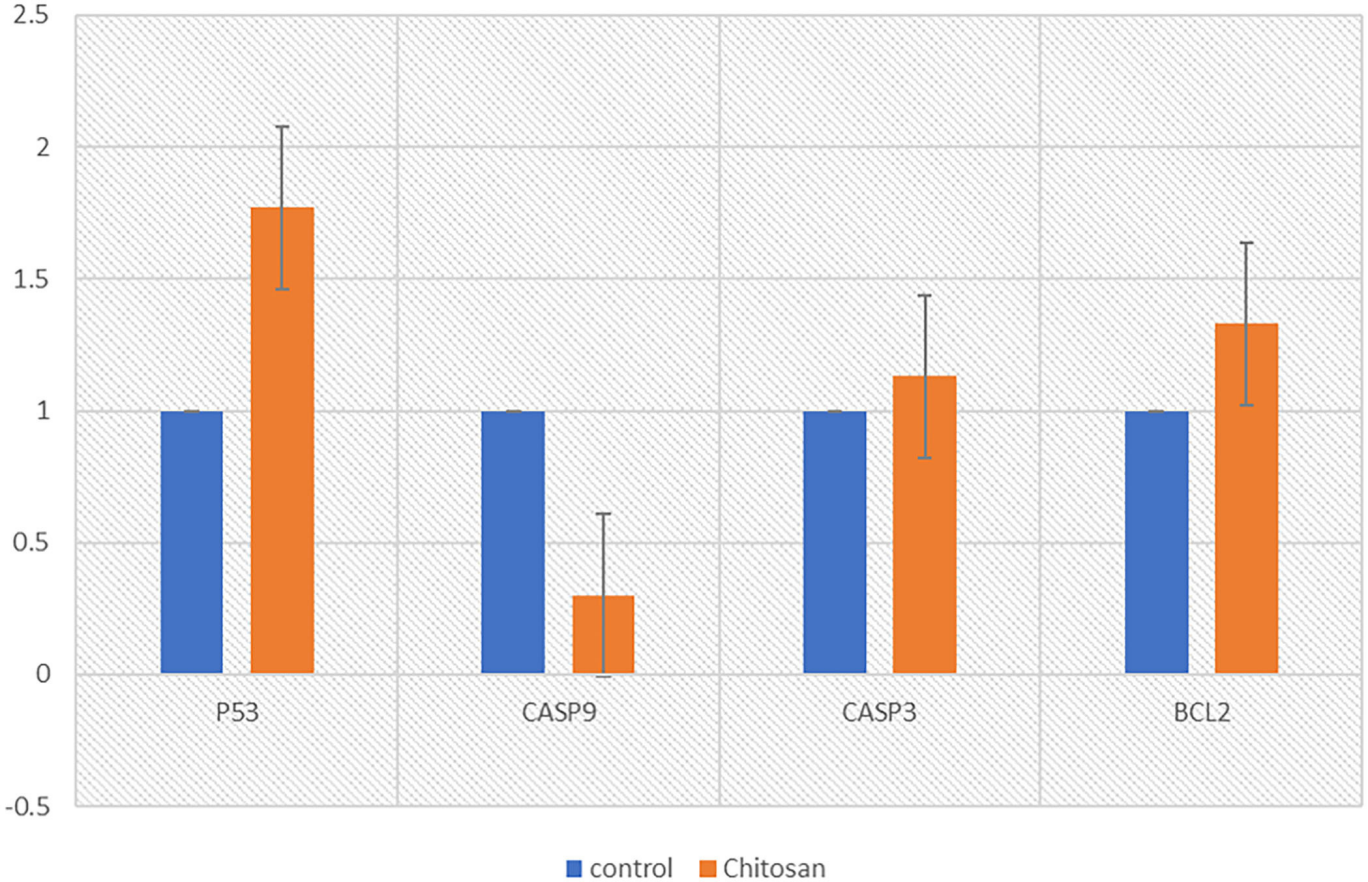
Chitosan effects on the P53, caspase-3, caspase-9, and BCL_2 mRNA levels in HeLa cells after treatment with chitosan for 24 h. The relative quantification of the target gene by the delta-delta-Ct method was done using the Qiagen software.

As shown in Figure 8, the expression of a different gene, the P53 gene, was increased (*p* < 0.05) in the chitosan treatment group in contrast with the control ones. while the expression of caspase-9 was reduced (*p* < 0.05).

## Discussion

Given the natural materials evaluation scenario as antibacterial and anticancer agents, the increase in antibiotic use has resulted in the emergence of antibiotic-resistant bacteria that have a dangerous impact on human health. Therefore, in this experiment, chitosan, graphene oxide, and GO:CS composite were used to estimate the antibacterial effect against BC and PAO1 by three different methods (turbidity measurement, agar diffusion method, and cell viability loss determination). Then, the superior antibacterial substance was evaluated as an anticancer agent. There are a few reports about the ratios of the GO:CS combination.

This study measured zeta potential analysis to confirm the surface decoration of CS, GO, and GO:CS groups. The zeta potential was negative at all pH values for GO due to the massive oxygen-containing sites on its surfaces (Cai et al., 2017). The negative charges of GO are a consequence of the ionization of the different groups present (Li et al., 2008), and the surface charge density should directly relate to the concentration of the ionized groups present at different pH values (Yan et al., 2009). When the pH is shifted to alkaline, the ionizable groups (carboxylic and/or hydroxyl group) on GO dissociate and GO gains its stronger negative charges.

Chitosan's zeta potential decreased linearly at pH levels ranging from 3 to 7, owing to protonation of the amino groups -NH2 to -NH3+. However, as the pH increases, the negative zeta potential does not decrease, indicating that the amino groups in chitosan are not deprotonated (Zhang and Bai, 2003).

Regarding the zeta potential of bacteria, the ZP technique may be interpreted as an indirect tool for determining the surface potential of bacteria, a physical feature that is essential for the maintenance of efficient cell activity. Therefore, in designing novel antimicrobials, it is essential to target the bacterial surface. It has been discovered that surface-acting drugs have an exceptional bactericidal effect and a low propensity to induce resistance. The ZP measurements may also be used to track alterations in the bacterial surface caused by several mechanisms (Maillard et al., 2021).

Jiang et al. (2013) indicated that the antibacterial impact of chitosan relies on different factors, including concentration, molecular weight, bacterial species used, test method, and solvents used. According to our data, with an increase in the CS concentration, a gradual decrease in the growth of the tested bacterial strains occurs; the concentration of chitosan also plays an essential role as an antibacterial agent (Goy et al., 2016). Additionally, the 43-kDa chitosan revealed higher effects than the 67-kDa chitosan vs. G– bacteria than G+ bacteria. In our study, chitosan has a different antibacterial effect against the G+ bacteria than the G– bacteria. According to our findings, the inhibition of chitosan carried out by the previous determination against BC was higher than PAO1 at all concentrations.

These findings are in agreement with No et al. (2002) and Jeon et al. (2001), who noticed that chitosan at a level of 0.1% (w/v) had more potent antimicrobial effects against G+ bacteria than G– bacteria. However, our study confirms the inhibition of chitosan on the G+ bacteria more than on the G– bacteria. Regardless of whether the effects of chitosan on G+ bacteria more than on G– bacteria or vice versa are somewhat controversial, it is essential to know how chitosan affects the bacteria. The antibacterial mechanism of CS may be because the reaction between the positive charge of CS and the negative charge of the bacterial membrane revealed in the lysis of the membrane results in the leakage of the cytoplasmic contents (Sundar et al., 2014), and thus the death of bacterial cells.

The previous findings did not show a specific role for GO; it could inactivate or stimulate bacterial proliferation without knowing the effect of GO on bacteria. Our results showed that when GO concentrations of 100 μg ml^−1^ to 300 μg ml^−1^ or 400 μg ml^−1^ were added, there was an increase in bacterial growth. These findings reveal that GO is a general growth enhancer but not a bactericidal or bacteriostatic agent. This result is consistent with Wu et al. (2018), who found that the GO does not inactivate bacterial growth and allows it to grow more than if it were seeded with only a nutrient. It is considered a general proliferation stimulator as a scaffold for microbial adhesion and diffusion, not as a bactericide or microbial agent. In contrast, GO had no significant impact on bacterial proliferation; it is not an inhibitor or stimulator of bacterial growth (Ruiz et al., 2011).

Inhibition of bacteria when using graphene is due to chemical contaminants present in graphene (Wong et al., 2014). In this study, with increased GO concentration, there was no change compared to control, or perhaps a slight inhibition with increased concentration to 700 μg ml^−1^ might be attributed to another unknown cause, but not to graphene. This result is also in harmony with Wu et al. (2018), who found that even at 200 μg ml^−1^, GO's biocompatibility remains functional, but it begins to reduce when the dose is about 300 μg ml^−1^. It is essential to mention that a high concentration of GO may be toxic to bacteria, but we want to inhibit bacteria by using low concentrations rather than high concentrations to avoid any damage.

Furthermore, the GO bactericidal features depend mainly on the purification and preparation assay due to pH and contamination of small molecules (Barbolina et al., 2016). In addition, the technology underlying the antibacterial effect of the GO nanosheets is not well understood. Different techniques are suggested for the antibacterial effect of graphene materials, such as direct contact mechanism, oxidative stress, and the trapping of microorganisms within the collected graphene nanosheets (Krishnamoorthy et al., 2011).

Mixing graphene with chitosan may suppress the toxicity of chitosan. In this study, when both bacteria were treated with GO-CS (2:1), the bacterial growth increased compared to the control and could be due to a higher GO ratio than CS, and GO prevented the inhibitory effect of CS. When treated with GO-CS (1:1) and GO-CS (1:2), different behaviors and inhibition of bacteria were observed, but these differences were minor compared to CS alone. The results indicate that the GO-CS combination is suitable for cell proliferation (Wu et al., 2018). Furthermore, Sundar et al. (2014) demonstrated that the GO-CS composite outperformed GO and CS in antibacterial activity.

Furthermore, at different concentrations, chitosan inhibits the growth and proliferation of the HeLa cell line and reduces cell viability (Li et al., 2019). Chitosan may directly fight tumor cells *via* interaction with cancer CM, extracellularly with a particular receptor, or endocytosis to induce cytotoxicity *in vitro* (Huang et al., 2004; Abedian et al., 2019).

Apoptosis is important in eliminating cancer cells that have mutated or overgrown. Various medicinal plants could stop tumor cell development *via* the initiation of apoptosis (Liu et al., 2015). This study revealed that chitosan enhanced early and late apoptosis and dead cells with decreased viable cells compared with the control HeLa cell line, indicating that it significantly stimulates apoptosis in HeLa cells (Chang et al., 2021).

Our results showed an elevation in the expression of p53 protein in HeLa cells reacting with chitosan. P53 is a tumor protein that acts as a cancer inhibitor, preventing tumor formation. It has three main actions, namely, growth inhibition, DNA repair, and apoptosis. The growth arrest prevents the cell cycle's progression, preventing damaged DNA replication. In this growth arrest, p53 may stimulate the transcription of proteins included in DNA repair. Apoptosis is the “last resort” to prevent the growth of cells with abnormal DNA. The cellular level of p53 must be tightly regulated. While it can inhibit cancer, too much p53 may enhance aging by too much apoptosis (Khazaei et al., 2017).

## Conclusion

The effect of chitosan against bacteria is under discussion, but it showed higher antibacterial activity against G+ than G– bacteria. Besides, it is edible and nontoxic for human health. Nevertheless, the argument about the antibacterial effect of graphene-based materials is lengthy because it depends on many factors. However, the results of this study support the view that graphene does not act as an antibacterial agent, so it may be used in biomedical nanotechnologies, such as facilitating surface-attached stem cells for orthopedics. Surprisingly, graphene mixed with chitosan inhibits the antibacterial effect of chitosan and the protection of the bacteria. Data revealed that the chitosan is stronger than the GO and GO:CS composites as an antibacterial substrate against bacteria. Furthermore, chitosan inhibited cell growth, proliferation, and viability while increasing cell apoptosis and dead cells in the HeLa cell line. The anticancer activity of CS promotes the function of the P53 protein (a tumor suppressor protein), inhibiting cancer growth. Finally, chitosan inhibits the growth of HeLa carcinoma cells by boosting the expression of the P53 gene.

## Data availability statement

The raw data supporting the conclusions of this article will be made available by the authors, without undue reservation.

## Author contributions

NAs, HE, AM, AS, BS, and ME-S: conceptualization and writing—review and editing. NAs, HE, AS, BS, and ME-S: methodology, formal analysis, and supervision. NAs, HE, AM, AS, BS, and ME-S: data curation, investigation, and methodology. AS and ME-S: methodology and software. NAl, GA, NB, MA, SS, MSA, MTA, MN, AS, BS, ME-S, NAs, HE, and AM: methodology and formal analysis. NAl, GA, NB, MA, SS, MSA, MTA, MN, AS, BS, ME-S, NAs, HE, and AM: formal analysis and resources. AM: resources, data curation, and investigation. NAs methodology and formal analysis. All authors contributed to the article and approved the submitted version.

## Funding

This work was funded by County Council of Västerbotten (BS), Cancer Research Foundation in Northern Sweden, Lion's Cancer Research Foundation in Northern Sweden (BS), and Kempestiftelserna (BS).

## Conflict of interest

The authors declare that the research was conducted in the absence of any commercial or financial relationships that could be construed as a potential conflict of interest.

## Publisher's note

All claims expressed in this article are solely those of the authors and do not necessarily represent those of their affiliated organizations, or those of the publisher, the editors and the reviewers. Any product that may be evaluated in this article, or claim that may be made by its manufacturer, is not guaranteed or endorsed by the publisher.
